# Segmental deformity markers offer novel indicators of deformity progression risk in deformity-matched adolescent idiopathic scoliosis patients

**DOI:** 10.1007/s43390-024-00927-7

**Published:** 2024-07-24

**Authors:** Fraser R. Labrom, Maree T. Izatt, Geoffrey N. Askin, Robert D. Labrom, Andrew P. Claus, J. Paige Little

**Affiliations:** 1 Biomechanics & Spine Research Group, Level 5, Centre for Children’s Health Research, Queensland University of Technology and Mater Health Services, 62 Graham St, South Brisbane, 4101 Australia; 2 Queensland Children’s Hospital and Mater Health Services, Brisbane, Australia; 3 Tess Cramond Pain and Research Centre, Royal Brisbane and Women’s Hospital, Brisbane, QLD Australia; 4 School of Health & Rehabilitation Sciences, University of Queensland, St Lucia, QLD Australia

**Keywords:** Adolescent idiopathic scoliosis, Progression, Three-dimensional, Magnetic resonance imaging, Segmental deformity

## Abstract

**Purpose:**

Identification of adolescent idiopathic scoliosis (AIS) patients with mild curvatures who pose significant risk of progressing to severe levels of curvatures is of paramount importance for clinical care. This study aimed to compare segmental deformity changes in AIS sub-cohorts that are dichotomised by progression status.

**Methods:**

Thirty-six female participants with Lenke 1 AIS curves were investigated with sequential MRIs during growth. Scans were reformatted to measure orthogonal segmental parameters, including sagittal/coronal wedging angles and axial rotation angles. Participants were dichotomised by progression. Two-tailed, independent sample t-tests were used to compare sub-cohort multi-segmental and segmental deformity parameters. Measurements were compared at each scan number and variable rates of change were determined using actual time between measures.

**Results:**

AIS progression status sub-cohorts were comparable at scan 1 for multi-segmental deformity parameters (e.g. major thoracic curve angle, rib hump, kyphosis) (*P* > 0.05). However, apical measures of coronal IVD wedging, axial IVD rotation and axial vertebral rotation were segmental parameters at scan 1 which were larger for participants whose AIS would later go on to clinically progress (all *P* < 0.05). Measures of segmental hypokyphosis were comparable between groups. As development was tracked at each subsequent scan, coronal and axial plane differences between groups increased in both magnitude and number of differences.

**Conclusion:**

Initial disparity and then subsequent increasing magnitude of change of axial rotation may indicate a higher propensity to clinically progress in the future. This knowledge hopes to provide useful management information for AIS care providers and prognostic education for patients alike.

**Level of evidence:**

II.

## Introduction

Despite adolescent idiopathic scoliosis (AIS) being typically characterised as a developing and evolving three-dimensional (3D) condition, curve progression is also highly variable [1]. A critical aspect of evidence based management of AIS patients is accurate prognostication of their expected curve progression. Adolescents with AIS who initially present with larger curves, greater skeletally immaturity, and/or pre-menarchal are known to be at a higher risk of deformity progression [2]. However, some patients possess stable curves despite having similar classical risk factors as other patients who progress to severe curves at rapid rates [3].

Of paramount importance to a treating orthopaedic practitioner, is the identification of AIS patients with minor curvatures who pose significant risk of progressing to moderate or severe levels of curvature [4, 5]. For moderate and severe curves, respectively, management typically includes conservative orthotic thoraco-lumbar bracing and serial radiographs [6], or operative interventions such as corrective fusion or growth preserving surgery [7, 8]. As such, early prognostication for these patients would evidently provide great benefit for guiding clinician management and patient/parent expectations. There has been a promising shift toward the analysis of the pathoanatomical variation of the AIS vertebral bodies (VBs) and intervertebral discs (IVDs) as it relates to AIS progression. There has been recent evidence that longitudinal coronal plane changes seen at the segmental level differ between dichotomised AIS cohorts whose curves either progressed or did not progress [9]. There remains a gap in the literature for this type of analysis in 3D however, as it is hypothesised that understanding the anatomical and biomechanical variation between vertebrae will elucidate pathoetiological mechanisms that drive progression of global 3D deformity [10]. These segmental deformity parameters may provide a source of prognostication for patients with minor global curvatures.

This study aims to compare 3D segmental deformity changes in AIS sub-cohorts that are dichotomised by participants whose curves do and do not clinically progress. This longitudinal prospective investigation offers the unique opportunity to analyse the differences observed within segmental scoliotic deformity between AIS participant sub-cohorts. It is hypothesised that axial rotational deformity parameters will uniquely differ between AIS participants who do and do not clinically progress.

## Methods

### Population

Study participants were sampled from an outpatient orthopaedic spine clinic from the Queensland Children’s Hospital, Brisbane, Australia. Participants with Lenke I [11] right-sided thoracic curves were recruited in accordance with inclusion criteria of Table 1. Routine review appointments in line with standard clinical practice, with clinical examination and serial standing radiographs, allowed measurement of scoliotic deformity and skeletal maturation. Rib hump measurements were acquired by experienced orthopaedic spinal surgeons, in accordance with previously validated protocols [12], via the Adam’s forward bend test with objective measurement via scoliometer to record the maximum angle of trunk rotation found in the thoracic region. The identification of the vertebral apex of the curve was not affected by physical examination, rather it was identified on standing radiographs as the most laterally displaced vertebra in the coronal plane. Scoliosis severity was assessed with reference to the International Scientific Society on Scoliosis Orthopaedic and Rehabilitation Treatment (SOSORT) 2016 guidelines, with mild curves classified by major thoracic Cobb angles between 10 and 20 degrees, moderate curves as between 21 and 40 degrees, and severe curves as greater than 40 degrees [13]. In line with standard clinical practice, participants were braced or unbraced at the discretion of their treating orthopaedic surgeons. Compliance of brace wearing was monitored at each review appointment. Participation within the study was ceased if participants received any form of surgical intervention. Participant and parental consent was gained in line with ethical approval granted from Mater Human Research Ethics Committee (HREC) (14/88/AM03), Queensland University of Technology HREC (1,200,000,281), and Children’s Health Queensland HREC (SSA/14/QRCH/411).

**Table 1:** Participant inclusion criteria

No	Criteria
1	AIS clinical diagnosis
2	Female sex
3	Aged 10yrs or older
4	Nil impairment of cognition
5	Risser grade < 2
6 7	Within six months of menarche or premenarchal Had not received any surgical spinal intervention

### MRI protocol

**Fig. 1:**
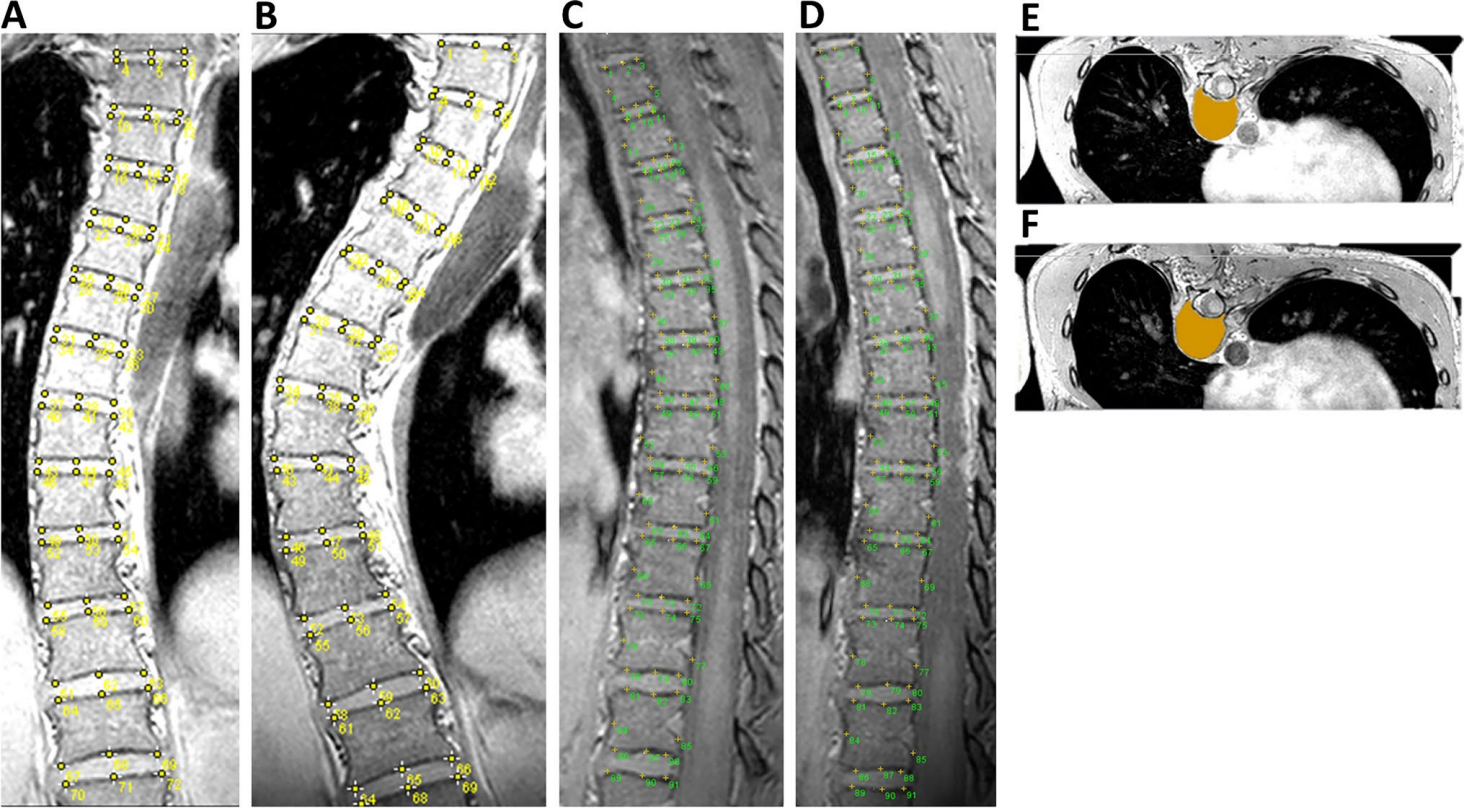
Orthogonal MRI reformatting images from final MRI scans of a non-progressive AIS curve (coronal Cobb angle = 23 deg; major thoracic kyphosis angle = 5 deg; rib hump = 13 deg) (Images A, C and E, respectively), and a progressive AIS curve (coronal Cobb angle = 64 deg; major thoracic kyphosis angle = 13 deg; rib hump = 18 deg) (Images B, D and F, respectively). Images A and B highlight coronal plane segmental anatomic markers. Images C and D highlight sagittal plane segmental anatomic markers. Images E and F highlight axial plane cuts at the curves’ apices, with an overlying annotation of the endplate surface area (blue)

At the conclusion of routine review appointments, the timing of which was determined at the discretion of treating orthopaedic surgeons, each participant received a supine thoracolumbar MRI. Ultimately, two to six MRIs for each participant were reformatted using image-processing software, ImageJ (v.1.45, National Institutes of Health, Bethesda, MD). This created orthogonal planar images which permitted subsequent measurement of spinal segmental structural morphology in 3D. This technique has been recently published [14] and the MRI acquisition protocol and image reformatting methods have been previously described [15–18]. Graphical reformatting examples are provided for each orthogonal plane with respective examples from each sub-cohort in Fig. 1.

### Segmental parameters

Wedging angles and rotational values were measured in accordance to previously described nomenclature [19], with intervertebral disc wedging (DW) and VB wedging (VBW) considered separately. Orthogonal segmental parameters were measured and respective definitions with descriptions of their positive direction of change are shown in Table 2. As previously suggested by the literature [20], these segments were considered in both the apical region (AR) and non-apical region (NAR). Within this investigation, the AR includes the vertebral apex, the articulating IVDs, and the immediately cranial and caudal VBs. The NAR includes all other VBs and IVDs included within the major curve that do not meet criteria for AR.

**Table 2:** Deformity parameter definitions for positive directions of change

Parameter	Definition
Coronal plane wedging	Increased wedging magnitude open to the curve’s convexity
Sagittal plane wedging	Increased wedging magnitude open to the curve’s posterior
Axial plane rotation	Increased rotation magnitude that directs the VB to the curve’s convexity, with the associated laminae displaced to the concavity
Axial plane torsion	Increased rotational magnitude of segmental elements that are rotated to the convexity more so in their cranial region than their corresponding caudal region

### Statistical analysis

Interobserver and intraobserver measurement error of these planar segmental parameters were measured and have been published for this cohort in previous works [15, 19]. SPSS Version 22.0 (IBM Corp, Armonk, NY) was utilised for all statistical analyses. Measurement normality was assessed with Shapiro–Wilk testing. Segmental parameter differences were compared using Mann–Whitney *U* tests or two-tailed, independent samples t-tests (with Welch’s correction if indicated) with a significance level of P < 0.05. Participants were dichotomised by progression status. Those participants who demonstrated an increase in major thoracic coronal Cobb angle of greater than five degrees (clinical measurement variability [21, 22]) between assessments or over the course of the study period were considered to have a progressive deformity. Those with equal or less than 5 degrees change in Cobb angle were considered to have a non-progressive deformity. Measurements were compared at each scan number and variable rates of change were determined using actual time between measures.

## Results

### Sub-cohort analysis of AIS patients stratified by clinical progression status

Participant progression status groups were compared at time of first MRI scan according to their growth parameters and global scoliotic markers (Table 3). Notably, those participants that went on to clinically progress were younger by 1.2 years (*P* = *0.025)* at scan 1, compared to those who did not clinically progress. This was also reflected in presenting earlier from their eventual onset of menarche, with both groups presenting before onset of menarche on average, but with progressive participants presenting on average 1.1 years (*P* = *0.016*) earlier than non-progressive participants. Despite this, both participant groups were statistically similar in growth metrics such as Risser grade and standing height at scan 1, as well as all traditional multi-segment scoliotic markers. When comparing participants with mild and moderate severity at scan 1, time to menarche, Risser grade and age were all comparable. The only deformity parameters which differed between the groups were mean apical coronal disc wedge angle (mild: -0.7(1.1) deg vs moderate: 3.4(4.1) deg; *P* = 0.008) and mean non-apical axial vertebral rotation (mild: -1.0 (0.7) deg vs moderate: -2.0(1.1) deg; *P* = 0.018).

**Table 3:** Growth and traditional multi-segment scoliotic markers at presentation (Scan 1) as stratified by clinical progression status

Parameters	Progressive	Non-progressive	*P*
N	24	12	-
Age (years)	12.5(1.2)	13.6(1.7)	0.025*
Menarche age (years)	-1.3(1.8)	-0.2(0.9)	0.016*
Risser Grade	0.5(0.7)	0.5(0.8)	0.876
Standing height (cm)	149.5(11.0)	156.4(9.5)	0.071
Major thoracic curve angle (^o^)	28.8(9.1)	24.6(8.2)	0.189
Rib hump (^o^)	10.1(3.4)	9.0(2.0)	0.221
Kyphosis (^o^)	− 10.9(6.4)	− 11.0(8.9)	0.978

^*^Denotes difference of means as significant at the 0.05 level (2-tailed)

Major thoracic curve angle statistically significantly differed between progressive and non-progressive groups at MRI scans 2, 3 and 4 with values of 35.6 vs 26.0 (*P* = 0.005), 41.0 vs 25.8 (*P* = 0.002), and 46.1 vs 23.5 (*P* = 0.038), respectively. Further comparisons were made using segmental deformity parameters at all scans (Table 4).

**Table 4:** Statistically significant segmental deformity parameters in coronal, sagittal and axial planes, observed between progressive and non-progressive participant groups

Scan	*N*_P_	*N*_NP_	Coronal	Sagittal	Axial
Scan 1	24	12	Apical sum coronal DW angle; **P = 4.20; NP = 1.09**	*Nil*	Mean major curve axial vertebral rotation; **P = 10.36; NP = 8.17** Mean apical axial discal rotation; **P = 12.52; NP = 9.63** Mean apical axial vertebral rotation; **P = 12.35; NP = 9.56**
Scan 2	24	12	Apical sum coronal DW angle; **P = 6.01; NP = 1.76**	*Nil*	Mean major curve axial discal rotation; **P = 10.92; NP = 7.84** Mean major curve axial vertebral rotation; **P = 11.36; NP = 7.89** Mean apical axial discal rotation; **P = 13.78, NP = 9.28** Mean non-apical axial discal rotation; **P = -2.42; NP = -1.39** Mean apical axial vertebral rotation; **P = 13.69; NP = 9.22** Mean non-apical axial vertebral rotation; **P = -2.33; NP = -1.33**
Scan 3	24	11	Sum coronal VBW angle; **P = 23.12; NP = 13.76** Apical sum coronal DW angle; **P = 7.77; NP = 4.06** Apical sum coronal VBW angle; **P = 15.61; NP = 9.38**	*Nil*	Mean major curve axial discal rotation; **P = 11.70; NP = 5.78** Mean major curve axial vertebral rotation; **P = 12.14; NP = 6.04** Mean apical axial discal rotation; **P = 14.99**; **NP = 7.52** Mean non-apical axial discal rotation; **P = -2.85; NP = -1.48** Mean apical axial vertebral rotation; **P = 15.05; NP = 7.49** Mean non-apical axial vertebral rotation; **P = -2.91; NP = -1.45**
Scan 4	18	2	*Nil*	*Nil*	*Nil*
Scan 5	4	1	*Nil*	*Nil*	*Nil*
Scan 6	3	0	*Nil*	*Nil*	*Nil*

All comparisons presented as means. All data presented is statistically significant to *P* < 0.05. “P” represents “Progressive”; “NP” represents “Non-progressive”; “DW” = ”Disc Wedge”; “VBW” = ”Vertebral Body Wedge”

### Rates of deformity progression by clinical progression status

**Fig. 2:**
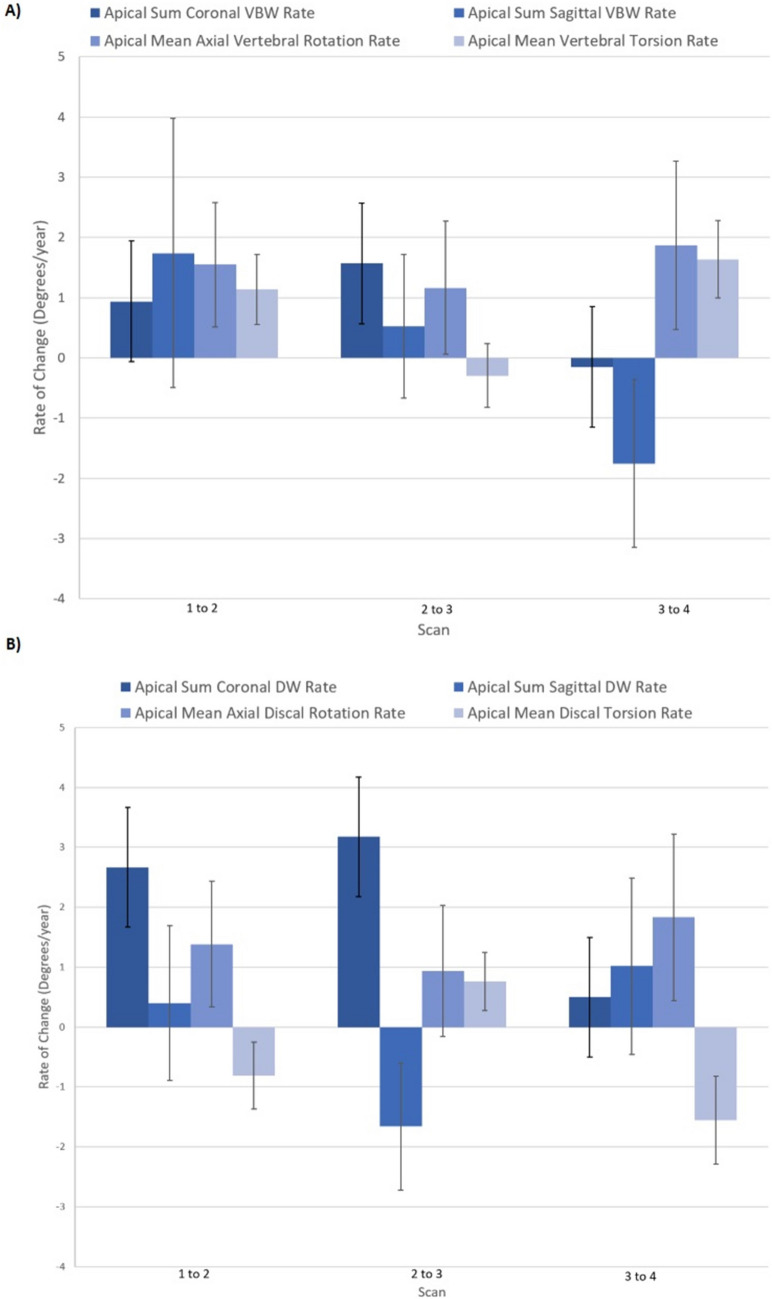
Segmental VB deformity rates of change stratified by time between successive MRI scan timepoints. Progressive cohort rates (**A**) and non-progressive cohort rates (**B**) of change are included. Data are presented as means (SE). Data pertaining to scans 5 and 6 were excluded secondary to low participant numbers

Rates of change for typical scoliotic markers and associated segmental apical parameters were also compared between the dichotomised groups. Comparisons of respective rate changes are made either between subsequent scans, or the overall rate between first and last scans obtained (Table 5). Rate change differences were mostly not statistically significant between clinically progressive and non-progressive groups. Rates of major thoracic curve angle (9.9(8.3) vs 0.8(2.2) degrees; *P* = 0.001) and rib hump (3.2(3.6) vs 0.7(2.5) degrees; *P* = 0.027) change showed statistically significant difference over the entire investigation course (first compared to last MRI scans) between progressive and non-progressive participant groups, respectively. Very few rate differences were captured between individual sequential MRI scans. Major thoracic curve angle rate of change differed between groups between scans 1 and 2 (11.9(13.5) vs -0.4(4.7) degrees; *P* = 0.006), while rib hump rate of change differed between groups between scans 2 and 3 (3.7 (5.5) vs -0.6 (4.9) degrees; *P* = 0.037). The only segmental deformity parameter rates of change which differed between groups between individual scans were sagittal plane measures. The apical sum sagittal VBW angle rate of change differed between groups between scans 1 and 2 [− 1.9 (7.7) vs 8.9 (18.7) degrees; *P* = 0.019], while the corresponding apical DW angle rate of change differed between scans 2 and 3 [− 0.3 (6.5) vs 4.7 (4.4) degrees; *P* = 0.025]. The segmental VB deformity rates of change are graphically represented in Fig. 2. The cumulative effect of these changes manifested as progressive cohort participants experiencing larger magnitudes of positively directed coronal wedging and axial rotation, and less sagittal kyphotic wedging.

**Table 5:** Statistically significant rates of change of scoliotic deformity parameters observed between progressive and non-progressive participant groups with sequential MRI scans

Rate Time Points	*N*_P_	*N*_NP_	Coronal	Sagittal	Axial
Scan F to L	24	12	Major thoracic curve angle rate; **P = 9.93; NP = 0.80**	Apical sum sagittal DW angle rate; **P = -0.63; NP = 4.48** Apical sum sagittal VBW angle rate; **P = 1.43; NP = 0.10** Non-apical sum sagittal DW angle rate; **P = -2.37; NP = 4.49**	Rib hump rate; **P = 3.19; NP = 0.72** Mean non-apical vertebral torsion rate; **P = -0.43; NP = 0.33**
Scan 1 to 2	24	12	Major thoracic curve angle rate; ***P* = 11.87; NP = -0.40**	Apical sum sagittal VBW angle rate; *** P* = -1.86; NP = 8.94**	*Nil*
Scan 2 to 3	24	11	*Nil*	Apical sum sagittal DW angle rate; **P = -0.27; NP = -4.70**	Rib hump rate; **P = 3.67; NP = -0.58**
Scan 3 to 4	18	2	*Nil*	*Nil*	*Nil*
Scan 4 to 5	4	1	*Nil*	*Nil*	*Nil*
Scan 5 to 6	3	0	*Nil*	*Nil*	*Nil*

All comparisons presented as means over the time between each identified scan. All rate data are presented in rate of degrees per year. All data presented are statistically significant to *P* < 0.05. “P” = “Progressive”; “NP” = “Non-progressive”; “DW” = ”Disc Wedge”; “VBW” = ”Vertebral Body Wedge”, “F” = ”First”; “L” = ”Last”

## Discussion

The progression of AIS curvature is variable in nature, with some patients possessing stable curves despite having similar risk factors at presentation as patients who progress at rapid rates [3]. This novel investigation of sequential MRI scans compared segmental deformity parameters between sub-cohorts whose curves did or did not clinically progress, despite being initially matched for the traditional multi-segment curve measures. The main findings demonstrated disparity between segmental parameters of the sub-cohorts on their initial scans. Then subsequent scans showed that those whose multi-segment measures clinically progressed had the greater propensity for increases in axial plane deformities. This suggests that segmental measures in the axial plane offer the strongest prospects to advance understanding of curve progression.

The group that went on to clinically progress was at first presentation both younger in chronological age by 1.15 years and had a younger menarche age that was on average 1.14 years earlier than non-progressive participants. Despite this, both participant groups were statistically similar in growth metrics at first presentation, such as Risser grade and standing height, as well as all traditional multi-segment scoliotic markers. The relevance of this must be highlighted, as the use of any subsequently identified differences in segmental parameters at the time of presentation between these two groups may prove useful as potential prognostic markers for curve progression risk. These main takeaways for respective dichotomies have been previously reported in the literature. Patients who initially present younger and/or are pre-menarchal are thought to have the greatest risk of deformity progression [2], due to periods of peak curve angle velocity occurring approximately one year pre-menarche [23]. Interestingly, measures of thoracic hypokyphosis at scan 1 were found to be comparable between both participant sub-cohorts. This supports the concept put forward in the wider research field that overall thoracic hypokyphosis may be a more generalised deformity observed across all severities of AIS rather than an indicator of progression risk at first clinical presentation [15, 24, 25]. Grivas et al. [26] suggested that a reduced thoracic kyphosis, by facilitating axial rotation, could be viewed as being permissive, rather than aetiological, in the pathogenesis of idiopathic scoliosis.

The apical measures of coronal IVD wedging, axial IVD rotation and axial vertebral rotation were particular deformity parameters at scan 1 which were larger for participants whose AIS would later go on to clinically progress. This corroborated with previous literature which has suggested that axial plane rotation is linked to progression of AIS curvature [27, 28]. In keeping with current findings of generalised thoracic hypokyphosis being present in the sagittal plane at scan 1, there were no segmental measures within the sagittal plane that differed between the progressive and non-progressive groups at initial presentation. Moreover, throughout the entire longitudinal investigation at every MRI scan time point, there were no statistically significant sagittal segmental parameters that differed between the sub-cohorts. As development was tracked at each subsequent scan, the coronal and axial plane differences between the groups increased in both magnitude and number of differences. For example, not only did the differences between sub-cohorts become more pronounced by pure measurement of angulation, but the differences began to be observed at multiple areas of the curve—across the entire major curve, apical and non-apical regions and within both VBs and IVDs. As suggested in previous bracing and modelling investigations [29–31], this morphological study uniquely adds to this growing body of scientific evidence by highlighting that segmental axial plane parameters are longitudinally related to coronal plane progression of scoliotic curves.

Rates of change of various scoliotic deformity parameters were compared within development phases for these sub-cohorts of participants to explore a potential sequence for deformity change. When examining the coronal plane mean rates of change of the summated apical VBW angles, there appeared to be little difference in rates between clinically progressive and non-progressive participants at initial scans. Sagittal plane mean rates of change observed for apical VBW angles demonstrated a definitive sequential pattern for clinically non-progressive participants. Initially, between scans 1 and 2, clinically non-progressive participants appeared to have a compensatory positive (that is, a locally kyphotic) change occurring at the apex in the VBs. This change was statistically significant with progressive participants having a -1.86 (7.72) degrees per year change (reducing kyphosis) in this early phase compared to an 8.94 (18.65) degrees per year change for the non-progressive participants. This initial reduction in kyphosis was observed in previous works on sequential deformity change [32, 33]. Axial plane segmental measure rates of change, despite trending towards favouring a positive correlation with progression status, did not reach statistical significance. Given there is a clear difference between groups at presentation, it highlights the importance of further axial plane deformity investigation in relation to curve progression prediction. This comparison of segmental deformity parameter rates of change in 3D is a novel investigation and a valuable addition to the field.

The current investigation does have limitations. Data drawn between later scans were often not statistically significant owing to under-powered participant numbers for this sub-cohort at these later scans. As such, there is an opportunity for further work on the topic as later timepoint changes may need further examination with greater statistical power to ascertain if these differences truly exist. Study generalisability is diminished as the selection criteria called for only Lenke I (primary single right thoracic) curves. While representing the most common type [34, 35], these findings may not be translatable to other varieties of curve types. Also, despite MRI becoming a more researched and validated approach for scoliosis imaging which correlates to upright imaging [36], it is reliance on supine posturing may decrease the magnitude of the more compressible and ductile IVDs compared to the rigid VBs [37]. The study protocol called for minimal disruption to standard clinical care of all participants, and as such, bracing did not exclude one from recruitment. This must be acknowledged as a limitation of the present study given the inherent biomechanical environment differences experienced by a cohort mixed with those braced and unbraced. Particularly, there is likely a pertinent limitation to the analysis of rotational or inter-vertebral measures given these are anticipated to be most affected by bracing treatment. However, the authors prescribe to the approach of pragmatic research design given this research is likely to impact clinicians managing the care of already braced individuals through outpatient clinics. These findings have furthermore been reproduced in other participant populations who were similarly braced throughout treatment [29–31]. Finally, the dichotomisation of participants into binary progressive/non-progressive classifications may inadvertently restrict the ability to analyse segmental deformities and their relations to scoliotic curvature—both of which are otherwise continuous variables. There is risk that binary categorisation fails to capture the continuum of developing deformity, especially around the pre-determined cut-off of five degrees of coronal Cobb angle measurement change. However, the application of well-reasoned dichotomous cut-offs of continuous variables is commonly required for clinical interpretation of diagnostic measures, as well as the subsequently informed clinical decision making. Current clinical practice uses this cut-off, which reflects the accepted measurement variability of Cobb angle measures [21, 22], to inform of clinically relevant curve progression, hence its inclusion within the current research protocol. Future research directions could include a detailed analysis of continuous variable correlations to establish a priori segmental deformity cut-offs, which are then validated in high-powered non-progressive and progressive cohorts to determine normally distributed values. The expansion of this topic, in conjunction with developing machine learning models targeted both at prognostic modelling and image reformatting from non-ionising MRI scans, could potentially provide a more robust and dynamic form of assessing progression risk in skeletally immature AIS patients.

Overall, this longitudinal prospective investigation offered the unique opportunity to analyse the differences observed within segmental 3D scoliotic deformities of AIS participant sub-cohorts. The highlighted concurrent coordination of coronal and axial plane change has been suggested to occur in progressive AIS patients within previous literature [27, 33], but the sequence and consistency of these changes have yet to be reported given only two comparative timepoints were previously utilised in these prior studies. Here, the current multi-timepoint study has demonstrated that initial disparity and then subsequent increasing magnitude of change of axial rotation may indicate a higher propensity to clinically progress in the future. The current investigation also expanded on previous evidence of generalised initial thoracic hypokyphosis within the wider AIS population [24, 27]. This longitudinal, 3D, segmental analysis of AIS by sub-cohort stratification is the first of its kind and creates a foundation for future research to investigate the sequential nature of the development of progressive AIS. This knowledge hopes to provide useful management information for care providers and prognostic education for patients alike.

## Data Availability

Research data supporting this publication are available via direct contact with the corresponding author.
